# Whole mitochondrial genomes unveil the impact of domestication on goat matrilineal variability

**DOI:** 10.1186/s12864-015-2342-2

**Published:** 2015-12-29

**Authors:** Licia Colli, Hovirag Lancioni, Irene Cardinali, Anna Olivieri, Marco Rosario Capodiferro, Marco Pellecchia, Marcin Rzepus, Wahid Zamani, Saeid Naderi, Francesca Gandini, Seyed Mohammad Farhad Vahidi, Saif Agha, Ettore Randi, Vincenza Battaglia, Maria Teresa Sardina, Baldassare Portolano, Hamid Reza Rezaei, Petros Lymberakis, Frédéric Boyer, Eric Coissac, François Pompanon, Pierre Taberlet, Paolo Ajmone Marsan, Alessandro Achilli

**Affiliations:** Institute of Zootechnics, Università Cattolica del S. Cuore, Piacenza, 29122 Italy; Research Center on Biodiversity and Ancient DNA – BioDNA, Università Cattolica del S. Cuore, Piacenza, 29122 Italy; Dipartimento di Chimica, Biologia e Biotecnologie, Università di Perugia, Perugia, 06123 Italy; Dipartimento di Biologia e Biotecnologie “L. Spallanzani”, Università di Pavia, Pavia, 27100 Italy; Institute of Food Science and Nutrition - ISAN, Università Cattolica del S. Cuore, Piacenza, 29122 Italy; Université Grenoble Alpes, Laboratoire d’Ecologie Alpine, Grenoble, 38041 France; Department of Environmental Sciences, Faculty of Natural Resources and Marine Sciences, Tarbiat Modares University, Noor, Mazandaran 46414-356 Iran; Natural Resources Faculty, University of Guilan, Guilan, 41335-1914 Iran; School of Applied Sciences, University of Huddersfield, Huddersfield, HD1 3DH UK; Agricultural Biotechnology Research Institute of Iran (ABRII), North Branch, Rasht, 41635-4115 Iran; Department of Animal Production, Faculty of Agriculture, Ain Shams University, Cairo, 11241 Egypt; Laboratorio di Genetica, Istituto per la Protezione e la Ricerca Ambientale (ISPRA), Bologna, 40064 Italy; Department 18/Section of Environmental Engineering, Aalborg University, Aalborg, DK-9000 Denmark; Dipartimento Scienze Agrarie e Forestali, Università degli Studi di Palermo, Palermo, 90128 Italy; Environmental Sciences Department, Gorgan University of Agriculture and Natural Resources, Gorgan, 49138-15739 Iran; Natural History Museum of Crete, University of Crete, Iraklio, Crete 71409 Greece

**Keywords:** Goat mitochondrial genome, mtDNA haplogroups, Domestication, Origin of *Capra hircus*, *Capra aegagrus*

## Abstract

**Background:**

The current extensive use of the domestic goat (*Capra hircus*) is the result of its medium size and high adaptability as multiple breeds. The extent to which its genetic variability was influenced by early domestication practices is largely unknown. A common standard by which to analyze maternally-inherited variability of livestock species is through complete sequencing of the entire mitogenome (mitochondrial DNA, mtDNA).

**Results:**

We present the first extensive survey of goat mitogenomic variability based on 84 complete sequences selected from an initial collection of 758 samples that represent 60 different breeds of *C. hircus*, as well as its wild sister species, bezoar (*Capra aegagrus*) from Iran. Our phylogenetic analyses dated the most recent common ancestor of *C. hircus* to ~460,000 years (ka) ago and identified five distinctive domestic haplogroups (A, B1, C1a, D1 and G). More than 90 % of goats examined were in haplogroup A. These domestic lineages are predominantly nested within *C. aegagrus* branches, diverged concomitantly at the interface between the Epipaleolithic and early Neolithic periods, and underwent a dramatic expansion starting from ~12–10 ka ago.

**Conclusions:**

Domestic goat mitogenomes descended from a small number of founding haplotypes that underwent domestication after surviving the last glacial maximum in the Near Eastern refuges. All modern haplotypes A probably descended from a single (or at most a few closely related) female *C. aegagrus*. Zooarchaelogical data indicate that domestication first occurred in Southeastern Anatolia. Goats accompanying the first Neolithic migration waves into the Mediterranean were already characterized by two ancestral A and C variants. The ancient separation of the C branch (~130 ka ago) suggests a genetically distinct population that could have been involved in a second event of domestication. The novel diagnostic mutational motifs defined here, which distinguish wild and domestic haplogroups, could be used to understand phylogenetic relationships among modern breeds and ancient remains and to evaluate whether selection differentially affected mitochondrial genome variants during the development of economically important breeds.

**Electronic supplementary material:**

The online version of this article (doi:10.1186/s12864-015-2342-2) contains supplementary material, which is available to authorized users.

## Background

The domestic goat (*Capra hircus*), which counts a worldwide population of more than 800,000,000 specimens and about 1200 breeds described (http://dad.fao.org), is among the “big five” livestock species defined by the Food and Agriculture Organization (FAO) [1] and is an invaluable source of milk, meat, skin and fiber for poor small holders and shepherds in developing countries and marginal areas [2].

On the basis of bone morphological changes associated with progressive taming [3], zooarchaeology suggests that goat domestication began about 11,000 years (ka) ago in an area stretching from the high Euphrates valleys in Southeastern Anatolia (Turkey) to the Zagros Mountains in Central Iran [4, 5] and located within the natural distribution of the wild ancestor species, the bezoar *Capra aegagrus* [6, 7]. This event is now considered as the final outcome of a gradual change from hunting to management of wild captive animals [3, 5, 8]. Due to their high rusticity and adaptability to harsh environments, goats represented a key resource during the Neolithic agricultural revolution and for the human migration waves that spread Neolithic culture out of the Fertile Crescent [9].

Previous studies of mitochondrial control-region haplotypes described six highly divergent haplogroups (Hg.s) in domestic goats: A, B, C [10], D [11], F [12] and G [13]. An additional haplogroup, named E, has been described by Joshi et al. [9] on the basis of two highly divergent control-region haplotypes. This was recognized as a sub-clade of haplogroup A when compared to a larger dataset [13]. The reported weak geographical structuring of goat mitochondrial variability was often interpreted as a consequence of the frequent transportation of goats along terrestrial and maritime routes of migration and commerce, probably during the early domestication phases [7, 10, 14, 15]. A subsequent comparison with wild stocks confirmed the presence of all “domestic” haplogroups in the current *C. aegagrus* populations, which could result from early translocations of animals and/or feralization before the worldwide spread of goats [7]. The haplogroup A is largely predominant (>90 %) among domestic goats, but rare (6 %) in the bezoar and never observed in the Iranian Zagros Mountains. The probable origin of haplogroup A occurred in Eastern Anatolia, where it is still present among wild populations, and its presence in Eastern Iran probably is the result of a subsequent feralization of domestic goats. The most frequent haplogroup in wild populations is C (39 %) detected in most of the bezoar distribution area and more common in Southern Zagros/Central Iranian Plateau. The evidence that C control-region haplotypes from Pakistan are the farthest from the domestic-related ones [7] disproved the Indus Valley domestication hypothesis suggested by archaeological remains from Mehrgarh (Baluchistan, Pakistan) [5]. Haplogroup F is still found in wild populations (from Northern Caucasus to lower Indus Valley), but it is very rare in domestic goats (<0.2 %), as it was identified only in three Sicilian samples [12]. The other haplogroups were found only in Iranian (D and G) or in both Northern Iranian and Eastern Anatolian bezoars (B). It has been proposed that these haplotypes might have entered the domestic goat gene pool either during the early spread of domestic goats, or due to small-scale domestication events. These findings indicate that the process of goat domestication occurred not only in Eastern Anatolia, as marked by haplogroup A and supported by zooarchaeological data [5, 8], but possibly also in Central Iran (Zagros Mountains and Iranian Plateau). This additional easternmost domestication event has been marked by haplogroup C, although it led only to a small contribution detectable in the mitochondrial gene pool of current domestic goats (1.5 %) and no archaeological substantiations [7].

MtDNA haplotypes belonging to haplogroups A and C have been found in ancient goat samples retrieved from an early Neolithic site in Southern France [16]. The two haplogroups occurred with almost the same frequency among the analyzed bones (i.e. 8 samples carrying A haplotypes and 11 samples carrying C haplotypes), suggesting that domestic goat populations were already characterized by the mtDNA variants A and C [16] during the first colonization waves that brought Neolithic farmers into the Mediterranean area about 7.5 ka ago [3].

Haplogroup divergence times calculated on different control-region datasets, usually by employing different calibration points, span from 100 to 940 ka [9–11, 17], thus largely predating the domestication events (~11 ka). These data suggest that many sub-haplogroups were already present among the bezoar populations and, therefore, that many lineages were included in the domesticated stocks. Previous studies on livestock species showed that phylogenies based on short mitochondrial sequences can be heavily affected by the confounding effects of homoplasies [18–21] and mitochondrial pseudogenes [22–24], which blur the real extent of lineage divergences/similarities. The available goat sequencing data are usually restricted to a few hundreds of control-region base pairs (bps), spanning from np 15431 to np 16643. Moreover, several complete goat mitochondrial genomes deposited in GenBank are probably chimeric or affected by NuMtS (i.e. nuclear sequences of mitochondrial origin) [22], including the previously adopted mtDNA goat reference sequence NC_005044 [22, 25]. In order to overcome these drawbacks, two recent papers have already extended the analysis to the mtDNA coding genes, but either failed to explore the complete mitochondrial molecule [17] or focused only on a new mtDNA haplotype [26].

We present the first extensive survey of the entire mitogenome variability based on 81 novel complete sequences from domestic goats (*n* = 76) and wild relatives (*n* = 5). This analysis allowed us to accurately define those (sub-) haplogroups involved in the domestication process(es), and to provide haplogroup coalescence estimates falling at the interface between Epipaleolithic and early Neolithic periods and expansion times falling into the Neolithic.

## Results

### The phylogeny of goat mitochondrial genomes

An initial collection of 758 mtDNA samples from *C. aegagrus* (*n* = 19) and *C. hircus* (*n* = 739; mostly from Western Eurasian breeds) was preliminarily characterized through control-region sequencing (Additional file 1: Table S1). This dataset, including 70 previously published samples [7], was used to build a haplotype network (data not shown). Overall, the network evidenced a high number of homoplasies and crosslinks, but it was useful to select 81 samples (from 76 domestic goats and five bezoars) for complete sequencing (Additional file 1: Table S2) using the criterion of including the widest possible range of mtDNA variation. The selected mitogenomes belonged to all known haplogroups, with few notable exceptions: i) the very rare haplogroup F, identified so far only in three domestic goats from Sicily [12], was not represented within our initial dataset of domestic goats; ii) the single control-region haplotype A in our *C. aegagrus* samples was re-classified as C by preliminary sequencing of some informative coding-region segments, which encompass diagnostic single nucleotide polymorphism (SNP) markers of A (at nps 3194/7839) and C (at nps 2885/3002/3131/3293/7657); iii) lastly, the few G control-region haplotypes were obtained from degraded DNA molecules, which allowed only partial coding-region sequencing that most likely confirmed the G affiliation (Additional file 1: Table S3). The 81 novel mitogenomes were compared with the revised goat reference sequence (GRS; NC_005044.2 – Additional file 1: Table S4). Various measures of molecular diversity were evaluated on the final dataset of 84 mitogenomes, which included 83 different haplotypes. After excluding ambiguous sites and indels (insertions/deletions), we identified a total number of 1003 variant sites (Table 1): 774 in the coding region (15414 nucleotides) and 229 in the D-loop (1213 nucleotides). Overall we observed an average number of 60.470 ± 16.628 nucleotide differences between two randomly chosen sequences. Figure S1 illustrates the distributions of nucleotide diversity (π) and total number of substitutions (continuous and dotted lines, respectively) along the mitogenome (Additional file 1: Figure S1). As expected, the highest diversity was observed around the HVS-I segment (hypervariable segment-I, from np 15,707 to np 16,187), with a peak of π = 0.059. The latter value is higher than those previously reported in horses [21]. Within the coding region, the highest number of variant sites was found in protein-coding genes (*n* = 655), mostly synonymous mutations. These data are consistent with previous studies on human and horse mitochondrial genomes [21, 27–29].

**Table 1:** Distribution and recurrence of mutations in the 84 goat mtDNA sequences

	CONTROL	CODING		
	D-loop	rRNA	tRNA	mRNA
Length in base pairs^a^	1213	2529	1513	11370
Invariable sites	984	2456	1467	10715
No. of variable sites	229	73	46	655
Proportion of variable sites	0.233	0.030	0.031	0.061
Sites with a single hit	96	50	36	470
Sites with two hits	25	10	4	51
Sites with three or more hits	108	13	6	134
Transitions	221	70	45	627
Transversions	12	3	1	28
Transition/Transversion ratio	18.4	23.3	45.0	22.4

^a^Lengths of protein-coding genes were readjusted and extended by considering the overlapping segments. Regarding the tRNA loci, the overlapping portions were counted only once

**Fig. 1:**
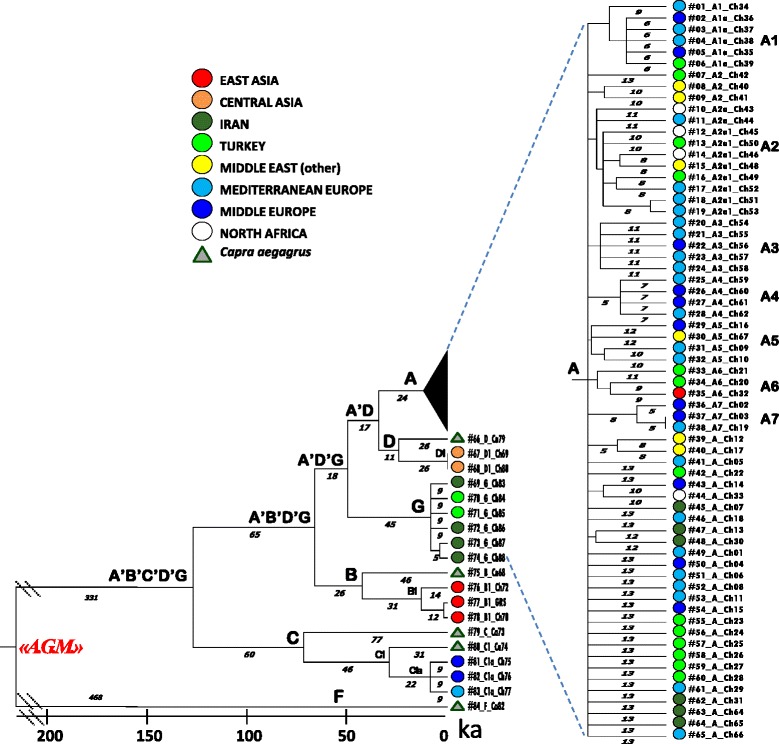
Schematic phylogeny of complete mtDNAs from modern domestic and wild goats. The tree encompasses 84 sequences and was rooted by using a published sheep (*O. aries*) sequence (not displayed). “A.G.M.” indicates the reconstructed Ancestral Goat Mitogenome. The topology was inferred by a maximum parsimony approach, while maximum likelihood (ML) time divergences based on synonymous substitutions are shown below the branches. The right inset shows the complete A branch rooted in D. Further details are given in Additional file 1: Table S4 and Figure S2

A parsimony approach was applied to infer evolutionary relationships from the final dataset of 84 complete mitogenomes. Eventually, only coding-region substitutions were included in the tree (Additional file 1: Figure S2) because of the extraordinary control-region variability (mainly around HVS-I, see Additional file 1: Figure S1) and high indels’ instability. The obtained topology confirmed all previously known control-region branches (A-G), but also revealed many different sub-branches, particularly within the major branch A. Seven novel sub-branches, named A1 to A7, were identified (at least three different haplotypes were required here to nominate a new clade, with the only notable exception of D1), all marked by coding-region transitions. In order to convert mutational distances into time over the entire mitogenome, the goat mtDNA sequences were compared with a published complete mitogenome (KF302445) from a Comisana sheep (*Ovis aries*) [30], used as an outgroup. Diverse maximum likelihood (ML) and Bayesian analyses were employed, as described in Materials and Methods. Initially, an ML tree based on synonymous mutations alone was estimated (Fig. 1). In fact, contrary to the number of potential factors causing time-dependent rates [31], synonymous mutations are virtually neutral and not subject to the effect of purifying selection, even though saturation might still be an issue with long time frames. Using CODEML and the mammalian mtDNA genetic code, we calibrated the synonymous molecular clock at 7.77 × 10^−8^ substitutions per year (at 3790 codons) or 1 substitution every 3397 years, after verifying the clock model hypothesis (*p-value* = 0.214). This mutation rate was also used to convert into time the rho estimates based on synonymous mutations (Table 2). The deepest node corresponds to the single Ancestral Goat Mitogenome (AGM), from which all modern goat mtDNA sequences derive, and was dated ~460 ka. The F bezoar mitogenome radiates first in the tree, then a major split (~130 ka ago) separates haplogroup C (dated ~80 ka) from the remaining mtDNA haplotypes. A subsequent branching separates two sister clades (B and A’D’G) both dated about 50 ka. Haplogroup B encompasses four samples, including GRS and one bezoar. The remaining wild samples belong to haplogroup D together with two domestic goats from Kyrgyzstan (with the same haplotype); affiliation to haplogroup D was also confirmed by considering recently published partial coding sequences [17]. The other bezoar mitogenomes, within haplogroups B and C, are ancestral to their most closely related domestic clusters B1 and C1a, dated 14.2 and 9.2 ka, respectively. These estimates are very similar to those of the A (12.8 ka) and G (9.0 ka) clusters, which include only *C. hircus* mitogenomes. In summary, all domestic clusters (A, B1, C1a and G) originated between 14 and 9 ka ago, as confirmed by both ML and rho statistics estimates (Table 2).

**Table 2:** Age estimates based on different datasets

	ML (synonymous sub.s)		Rho (synonymous sub.s)		ML (all substitutions)^a^		ML (only coding region)		Rho (only coding region)	
Node	T(ka)	ΔT(ka)	T(ka)	ΔT(ka)	T(ka)	ΔT(ka)	T(ka)	ΔT(ka)	T(ka)	ΔT(ka)
*A.G.M.* ^b^	467.7	59.2	457.9	37.3	652.0	34.0	734.9	61.1	715.5	51.2
A’B’C’D’G	136.7	21.3	125.7	17.4	186.6	12.2	214.3	24.0	187.4	23.0
A’B’D’G	71.2	12.2	57.3	10.6	115.9	8.3	124.8	15.6	95.6	14.9
A’D’G	53.5	9.8	42.5	8.8	89.3	6.8	93.1	12.8	68.5	12.0
A’D	36.9	8.1	25.4	5.8	66.0	5.9	63.6	11.3	42.0	8.1
A	12.8	1.9	14.1	1.4	23.4	1.4	23.0	2.2	23.7	1.9
A1	9.2	2.8	10.2	3.5	20.9	1.9	18.6	3.5	19.2	4.9
A2	12.8	2.9	16.2	3.9	19.7	1.4	21.0	2.5	26.3	4.9
A3	10.7	1.9	15.6	3.3	19.7	1.5	19.5	2.7	24.7	4.5
A4	7.4	2.5	6.8	2.4	16.8	1.9	16.1	3.9	14.4	3.9
A5	12.8	3.7	12.7	3.5	21.5	1.8	19.9	3.4	19.6	4.7
A6	11.2	2.1	15.9	4.5	17.1	1.9	20.4	3.0	24.7	6.1
A7	4.8	4.2	3.4	4.4	11.1	1.9	9.3	4.0	6.9	3.6
B	45.7	9.4	47.6	9.1	70.9	6.2	76.7	11.9	78.3	12.7
B1	14.2	5.1	12.5	4.4	23.9	3.4	24.9	7.1	23.4	6.9
C	77.2	14.1	78.1	12.4	120.1	9.1	122.3	16.9	114.5	16.2
C1	31.4	7.6	31.4	7.3	54.3	5.2	51.6	10.1	49.4	9.8
C1a	9.2	3.4	9.1	3.2	15.9	2.2	17.4	5.0	16.5	4.7
D	26.3	7.2	32.8	7.8	45.2	5.6	49.4	10.9	60.4	11.8
D1	0.0	11.0	0.0	5.1	0.0	7.9	0.0	16.5	0.0	6.2^c^
G	9.0	2.7	9.1	2.5	23.1	2.2	22.1	4.6	22.7	4.3

^a^The entire genome was partitioned into six datasets: 1st, 2nd and 3rd positions of the codons, RNAs, HVS-I and the remainder of the control region

^b^Ancestral Goat Mitogenome

^c^A 95 % C.I. for the age of D1 is 0 to ln(20)/n in units of 4120 years

**Fig. 2:**
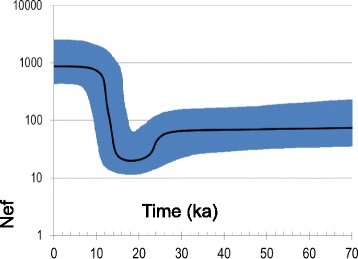
Bayesian Skyline Plot showing the goat population size trend with a generation time of 4.5 years [60]. The Y axis indicates the effective number of females. The thick solid line is the median estimate and the grey shading shows the 95 % highest posterior density limits. The time axis is limited to 100 ka, beyond that time the curve remains linear

**Fig. 3:**
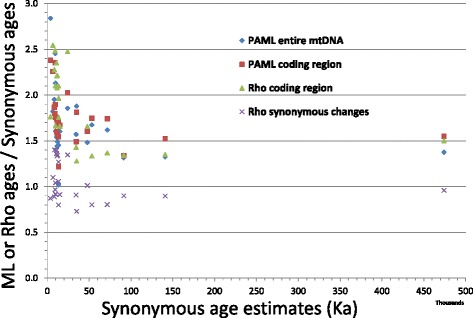
Comparison of ML and rho-statistics ages relative to the ML synonymous estimates. See Table 2 for further details

A Bayesian Skyline Plot (BSP) analysis was carried out to assess population expansions. The overall BSP points to a steep increase of the female effective population size about 12–10 ka ago (Fig. 2). This analysis was performed on the complete molecule after establishing six different partitions (1^st^, 2^nd^ and 3^rd^ codon positions, RNA genes, HVS-I and other control-region segments). The same partitions were also considered to perform ML analyses on the entire mitogenome. The final outcomes were an overall mutational rate of 3.95 × 10^−8^ substitutions per nucleotide per year (1 mutation every 1522 years) on the entire molecule and 1.57 × 10^−8^ substitutions per nucleotide per year (or 1 mutation every 4120 years) on the coding region alone. Both analyses revealed that the molecular clock could not be rejected (*p-values* > 0.05) when employing the complex GTR/REV model. A clear sign of purifying selection is apparent when calculating the non-synonymous/synonymous ratio, i.e. (ɷ) = 0.190 (Table 3). As expected, this ratio is significantly lower (*p-value* < < 0.001; Fisher’s exact test) in the deep portion of the tree and the comparison among domestic branches reveals slightly significant differences (*χ*2 *p-value* = 0.026) (Table 3); in particular the ɷ ratio of A is much higher than previously reported (ɷ = 0.049) [17]. Similarly, age values of younger clades are much higher when considering the entire panel of mutations rather than those based on synonymous changes only (Fig. 3), probably because the effect of purifying selection is incomplete and all the negatively selectable characters are still included [29].

**Table 3:** Rates of non-synonymous/synonymous differences (dN/dS) on the goat phylogeny

	dN	dS	dN/dS
Entire Phylogeny	114	600	0.190
Pre-domestic branches	46	361	0.127
Domestic branches	68	239	0.284
Comparisons within domestic haplogroups			
A	46	199	0.231
B1	3	9	0.333
C1a	3	8	0.375
D1	4	9	0.444
G	12	14	0.857

### Geographic distributions of goat mtDNA haplogroups

**Fig. 4:**
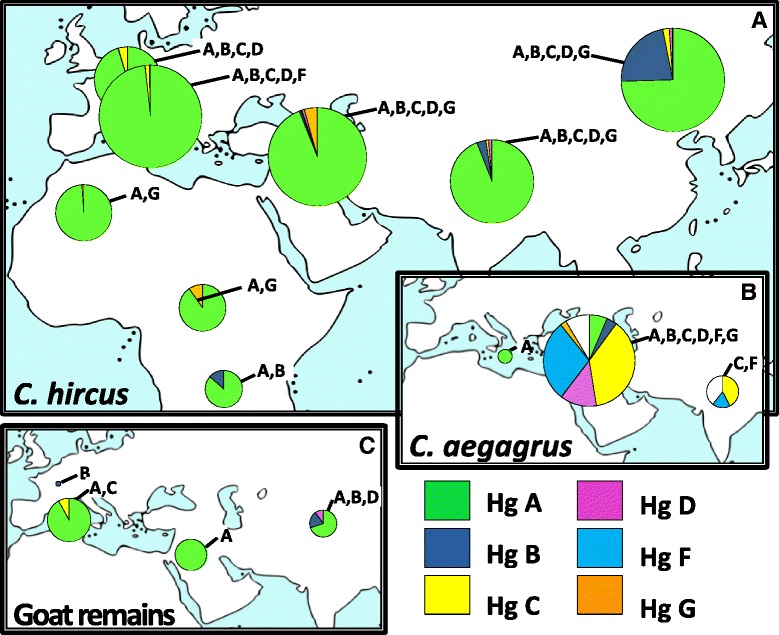
Spatial frequency distributions of goat mtDNA haplogroups in different geographic areas based on different datasets: modern breeds (*C. hircus*) **a** *;* wild goats (*C. aegagrus*) **b**; and ancient goat remains **c**. See Additional file 2 (Table S5) for more information

An analysis of the geographic distribution of goat haplogroups in Eurasia and Africa (Fig. 4), based on the control-region data currently available in literature or deposited in GenBank, confirms an overwhelming predominance of haplogroup A (~90.5 %) among domestic goats all over the world (including the Americas, Additional file 2: Table S5). The second most frequent haplogroup is B (~6.5 %), more common in Asia and Southern Africa, but also present in Europe. Haplogroup G (0.9 %) is restricted to North-central Africa and Asia, while the presence of haplogroups C and D is limited to Eurasia with an average frequency of 1.4 % and 0.6 %, respectively. Finally, haplogroup F was identified only in three Sicilian domestic samples. The bezoar samples analyzed so far are mainly from the Middle East (Iran, Turkey and Jordan) and South Asia (India, Pakistan and Bangladesh), where they are mostly characterized by the occurrence of haplogroups C (37.4 and 42.4 %) and F (28.9 and 18.2 %). *C. aegagrus* is also represented by haplogroups D (12.9 %), A (6.4 %), B (3.7 %) and G (2.2 %) in the Middle East. Wild goats are also found on some Mediterranean islands (*C. aegagrus cretica*), even though the widely accepted opinion is that they derive from the feralization of very early domestic animals [32]. Finally, the few ancient mtDNA haplotypes (Additional file 2: Table S5) were found in goat remains excavated in Central/East Asia (A, B and D), the Near East (only A) and Europe (B in mainland; A and C in the Mediterranean area). The presence of A and C in the Mediterranean area since ancient times could be due to the Neolithic spread attested by the first appearance of Cardial pottery in the Eastern Adriatic since 8.5 ka ago [33–35].

## Discussion and conclusions

### Domestication of *C. aegagrus*

Wild goats were widely distributed throughout Southwestern Eurasia during the Middle Paleolithic (300 to 45 ka ago). In an attempt to reconstruct past demographic histories based on phylogenetic inferences, the tree in Fig. 1 suggests that a drastic bottleneck occurred during the Late Eurasian Saalian glacial (~130–160 ka ago) [36–39], which is compatible with the age of the major macro-haplogroup A’B’C’D’G. During the following Riss-Würm interglacial relapse (~115–130 ka ago), the A’B’D’G lineages evolved and expanded independently from the C-derived populations. Many lineages did not survive the following Würm glacial period (~12–71 ka ago) and the drastic climatic changes during the Last Glacial Maximum (LGM, ~19.5–25 ka ago), but some of them, corresponding to the principal goat haplogroups, were preserved in the Near East refuge areas [40], survived the severe drop in temperature known as the Younger Dryas (~11.5–12.7 ka ago) [41, 42], and provided the necessary substrate of variability for later goat domestication and management in an area from the Zagros Mountains to Southeastern Anatolia, as testified by the abundance of goat remains in Neolithic sites from that regions [8, 43].

The main goat haplogroups were identified through control-region analyses following an approach similar to that adopted for other domesticated species, such as sheep [3, 30, 44–46]. However, in the case of both horses and bovines, the mtDNA haplogroup classification received a major makeover from the exploration of the entire mitogenome variability [21, 47–49]. A common feature of all livestock phylogenies is that the control-region molecular clocks turned out to be very inadequate for an accurate dating of mitochondrial lineage divergences. When mtDNA protein-coding genes were considered [17], goat haplogroups were dated to the Middle Paleolithic, thus suggesting that multiple related lineages were domesticated ~11 ka ago in an area spanning from Southeastern Anatolia in Turkey to Zagros Mountains in Iran, by incorporating pre-existing variation [7–9, 17]. This domestication center, further confirmed by zooarchaeological data [43], left a clear signature in the mtDNA gene pool of current domestic breeds, as attested by the large predominance of A haplotypes. Our analyses allowed us to assess the coding-region variability within this clade (Additional file 2: Table S6) and to date for the first time this major goat haplogroup to the Epipaleolithic period; a few A sub-haplogroups were also phylogenetically defined and dated to the early Neolithic. The implication is that more than 90 % of current domestic goats might descend from a single foundress, represented by the internal node A in our phylogeny. Obviously, the absence of A bezoars in our dataset is an issue, as well as the long-term calibration point, which might represent an underestimate of the true sheep/goat divergence. Thus, by also considering the alternative hypothesis of many founder lineages within haplogroup A independently proposed by Naderi et al. [7] and Nomura et al. [17], we might suggest that at least seven of them have been identified in our dataset as represented by the ancestral mitogenomes of sub-haplogroups A1–A7.

Moreover, in some instances, our phylogenetic reconstruction clearly shows that other domestic clusters are nested within wild branches and each of them descends from a unique wild haplotype. Therefore, excluding the rare but possible occurrence of stochastic backcrossing between wild and domestic animals, the first domestication process probably involved only four additional female goat populations corresponding to one founder haplotype for each of the other domestic lineages, i.e. B1, D1, C1a and G,. We cannot exclude that, when analyzing further *C. aegagrus* samples at the maximum level of resolution, several additional sub-groups could be identified within each haplogroup, as previously discussed for the A lineages. Likewise, the possibility that some of the mitochondrial haplotypes belonging to lineages B1, D1 and C1a and presently found in domestic animals might derive from introgression of wild lineages cannot be completely ruled out. Yet, events of introgression from wild animals into domestic herds are incidental and usually male-mediated [50], and they were not identified so far even when analyzing a larger dataset of wild and domestic control-region sequences [7, 13]. We have also verified that all available control-region haplotypes (including those from the present study) belonging to haplogroups B, C and D could be specifically assigned either to wild or domestic branches as expected from individual phenotypes (i.e. wild or domestic), without finding any notable exceptions.

The Bayesian analysis of our goat sequences also shows that, after domestication, the early domestic populations soon experienced a drastic demographic expansion about 12–10 ka ago (Fig. 2). However, since our tree includes samples from a very wide geographical area, we cannot exclude that additional and perhaps more recent signals of local demographic expansions could be detected by analyzing larger and more geographically structured datasets, particularly along the domestication routes.

A more fascinating scenario could also be envisioned for haplogroup C since this haplogroup might represent a marker of a concomitant secondary domestication, as already suggested by control-region analyses [7]. To support this scenario, our age estimate for C1a is compatible with the first domestication wave and, more importantly, the C clade represents an independent branch in our tree, which is separated from the A’B’D’G cluster by over one hundred thousand years, thus suggesting a genetically distinct population origin.

### The prevalence of haplogroup A: a close similarity between cattle and goat domestication

Phylogenetic analyses based on entire mitogenomes revealed that, similarly to horses and taurine cattle, the ancient goat populations might have passed through a severe bottleneck during the Late Saalian glacial maximum with a single sequence (A’B’C’D’G) from that period as the common ancestor of most goat mitogenomes. However, the recent goat evolutionary history suggests more similarities with *Bos primigenius* domestication (Additional file 2: Table S7). Present-day cattle and goat mitogenomes belong to very few haplogroups, with most of them (>85 % in Eurasia) falling within a single branch (T3 and A, respectively), whose sequence coalescence time corresponds to a very similar time estimate of about 11–13 ka. As in the case of the cattle T3 branch, this study and other published data support a Neolithic origin for all goat A mitogenomes (possibly differentiated into some sub-haplogroups, e.g. A1–A7) from a Middle Eastern bezoar population. At least four other minor lineages (B1, C1a, D1 and G) directly related to bezoar ancestors (i.e. B, C1 and D) were domesticated. However, unlike the bovine minor clades P and Q that suggest a possible European domestication or introgression, the geographical distribution of the low-frequency goat clusters indicates that secondary domestication *foci* were located in the same area between Anatolian Turkey and Iran. This leaves open the possibility of a more eastward Iranian center involving the furthest phylogenetically-related haplotype C1. Moreover, the new coding-region diagnostic mutational motifs defined in the present study (Additional file 2: Table S6) could be employed to revise phylogenetic relationships between modern breeds and ancient remains with the overall objective of testing the proposed scenario of secondary early domestication processes that took place before the occurrence of morphological modifications, and evaluating whether selection differentially affected mitogenome variants during the development of economically important breeds.

## Methods

### Sequencing of goat mitochondrial genomes

All experimental procedures were reviewed and approved by the Animal Research Ethics Committee of the University of Pavia, Prot. 2/2010 (October 15th, 2010), in accordance with the European Union Directive 86/609. Prior to the sequencing of entire mtDNA molecules, a preliminary sequence analysis of the control regions was performed. This allowed for the selection of 81 samples with good quality DNA and encompassing the widest range of mutational motifs. During the first phases of the experimental work we used the established Sanger sequencing approach rather than new sequencing technologies since, at that time, the latter did not yet guarantee complete coverage and could be still prone to artificial ambiguities [51]. The sequencing protocol was similar to those previously and successfully used for human and livestock mitogenomes [21, 30, 47, 52]. The oligonucleotides used to amplify and sequence the goat mitochondrial genome are reported in Additional file 2: Table S8. They were checked through GenBank BLAST to avoid amplification of nuclear insertions of mitochondrial sequences (NuMtS) [53]. In the last phases of the experimental work, thanks to the acquired affability and accuracy of the next generation techniques, additional 28 samples were amplified with the same oligonucleotide pairs, pooled together (5 μg of DNA in total) and sequenced on the Illumina Genome Analyzer IIx, platform at the IGA Technology Services, Udine, Italy.

Several parameters of the mtDNA sequence variation were estimated by using DnaSP 5.1. The variation of nucleotide diversity (π) along the entire mtDNA was estimated by assessing windows of 100 bps with step size of 50 bps centered at the midpoint. For an estimation of the synonymous/non-synonymous sites we created an alignment containing only the protein-coding genes, with the ND6 gene adjusted to present the same reading direction as the other genes. The “stop codons” were excluded from the analysis. Overlapping loci were counted twice leading to a final alignment of protein-coding genes equal to 11370 bps.

### Phylogeny construction and molecular divergence

The phylogeny construction was performed as described elsewhere [21, 30, 47, 52] and confirmed using an adapted version of mtPhyl 3.0 for a maximum parsimony (MP) analysis [54]. The modified .txt files are available upon request.

We constructed an MP tree including 84 goat mitogenomes (without D-loop) rooted on sheep, *O. aries*, mitogenome (KF302445). A first maximum likelihood (ML) analysis was performed using PAML X [55] by considering only the protein coding genes (and synonymous mutations). As already mentioned, the ND6 gene was reverse-complemented to present the same reading direction as the other genes and, under the vertebrate mitochondrial genetic code, the non-synonymous substitutions were excluded from the alignment and replaced with the ancestral base pairs. The “stop codons” were excluded from the analysis. Lengths of protein-coding genes were readjusted and extended by considering the overlapping segments. The final alignment (11370 bps long) was analyzed with CODEML, which calculates a synonymous mutation rate taking into account the mitochondrial genetic code. The second survey was carried out by considering six partitions in the molecule: one corresponds to the RNA genes (tDNA and 12S/16S rDNA), one to each codon position of the protein-coding genes (CDS), one to HVS-I, and one to the remaining D-loop sequences. In the final alignment, the rDNA and tDNA, CDS, HVS-I and D-loop segments were 4042 (overlapping sites between rDNA and tDNA were counted once), 11370 (see above), 481 and 732 bps long respectively. The non-coding region between np 5160 and np 5191 was not considered, but we checked that it was invariable. The best model able to describe the phylogenetic relationships among taxa was selected by using jModelTest [56]. Eventually, separate analyses were performed on the coding region and on the entire mitogenome by assuming a GTR/REV mutation model, a molecular clock and gamma-distributed rates, approximated by a discrete distribution with 32 categories. In order to check the clock hypothesis, likelihood ratio tests were applied with and without molecular clocks. The ML estimates were also compared with those directly obtained on the MP trees by using mtPhyl as averaged distances (ρ) of the haplotypes of a clade to the respective root haplotype, also known as rho-statistics [57], accompanied by a heuristic estimate of the standard error (σ).

We also obtained a Bayesian skyline plot (BSP) [58] from the goat phylogeny using BEAST 2.2.1 software [59]. We run 10,000,000 iterations with samples drawn every 5000 steps and used a generation time of 4.5 years [60]. BSPs provided a good visualization of the increase in diversity in the tree by estimating effective population sizes through time.

### Calibrating the goat mtDNA molecular clock

For the calibration point in the maximum likelihood analyses, we assumed an estimated bifurcation time between sheep and goat of 6,000,000 years (assuming a 95 % interval of 5–7,000,000 years in the BEAST analysis) based on fossil evidence as already used by Sultana et al. [11]. Internal calibration points were not available. Considering the time-dependency of molecular rate estimates [31], the use of a paleontological calibration point means that we are prone to possible biases mainly generated by non-synonymous substitutions and mutations affecting tRNA/rRNA genes (purifying selection) and the control region (tendency to saturation due to the high evolution rate). This long-term calibration point probably represents an underestimate of the true sheep/goat divergence and might have had a considerable impact on the date estimates.

### Availability of supporting data

Sequences of the novel goat mitogenomes have been deposited in GenBank under accession numbers KR059146 - KR059226 (81 complete mtDNAs) and KR059227 - KR059851 (625 mtDNA control regions). Phylogenetic data have been deposited in TreeBase (http://purl.org/phylo/treebase/phylows/study/TB2:S18595).

## Additional files

Additional file 1:
**Table S1.** Sources for the 758 goat control-region sequences. **Table S2.** Control-region haplotypes and haplogroup classification of the 758 mtDNA sequences from *Capra aegagrus* (*n* = 19) and *Capra hircus* (*n* = 739). **Table S3.** Partial coding-region haplotypes and haplogroup classification of two bezoar mtDNAs. **Table S4.** Source and haplogroup affiliation of the goat complete mtDNA sequences. **Figure S1.** Nucleotide diversity and total number of substitutions along the entire mtDNA. **Figure S2.** A putative most parsimonious tree of 84 complete mtDNA sequences from goats. (XLSX 1268 kb) Additional file 2:
**Table S5.** Goat haplogroup frequencies based on modern and ancient control-region mtDNA data from this study and downloaded from GenBank^a^. **Table S6.** Diagnostic mutational motifs of goat mtDNA haplogroups and sub-haplogroups. **Table S7.** A comparison of the phylogeographic features of goat, taurine and horse mtDNA haplogroups identified by analyzing domestic breeds from Eurasia. **Table S8.** Oligonucleotides used to amplify and to sequence (Sanger method) the goat mitochondrial genome. (PDF 652 kb)

## Abbreviations

AGM: Ancestral Goat Mitogenome
BEAST: Bayesian Evolutionary Analysis Sampling Trees
BLAST: Basic Local Alignment Search Tool
Bps: Base pairs
BSP: Bayesian Skyline Plot
CDS: Coding DNA Sequence
D-loop: Displacement Loop
dN/dS: Non-synonymous/synonymous ratio
DnaSP: DNA Sequence Polymorphism
FAO: Food and Agriculture Organization
GRS: Goat Reference Sequence
GTR/REV: General Time Reversible
Hg: Haplogroup
HVS-I: Hypervariable segment-I
Indels: Insertions/deletions
Ka: Kilo annos
LGM: Last Glacial Maximum
ML: Maximum Likelihood
mtDNA: Mitochondrial DNA
ND6: NADH, ubiquinone oxidoreductase core subunit 6
NuMt: Nuclear Mitochondrial DNA
PAML: Phylogenetic Analysis Using Maximum Likelihood
rDNA: Ribosomal DNA
rRNA: Ribosomal RNA
SNP: Single Nucleotide Polymorphism
tDNA: Transfer DNA
tRNA: Transfer RNA
