# Supplementary material for: Whole mitochondrial genomes unveil the impact of domestication on goat matrilineal variability
Source: BMC Genomics. 2015 Dec 29;16:1115. doi: 10.1186/s12864-015-2342-2 (PMC4696231; doi:10.1186/s12864-015-2342-2)
Supplement: Additional file 1: — Table S1. Sources for the 758 goat control-region sequences. Table S2. Control-region haplotypes and haplogroup classification of the 758 mtDNA sequences from Capra aegagrus (n = 19) and Capra hircus (n = 739). Table S3. Partial coding-region haplotypes and haplogroup classification of two bezoar mtDNAs. Table S4. Source and haplogroup affiliation of the goat complete mtDNA sequences. Figure S1. Nucleotide diversity and total number of substitutions along the entire mtDNA. Figure S2. A putative most parsimonious tree of 84 complete mtDNA sequences from goats. (XLSX 1268 kb) [file 12864_2015_2342_MOESM1_ESM.xlsx]

Table S5. Goat haplogroup frequencies based on modern and ancient control-region mtDNA data.[a]

Domestic Breeds

	Hg A	Hg B	Hg C	Hg D	Hg F	Hg G	Other	Total	References
**Africa**	**574 (95.19)**	**14 (2.32)**	**…**	**…**	**…**	**15 (2.49)**	**…**	**603**	
Northern	383 (98.97)	…	…	…	…	4 (1.03)	…	387	Amills Direct subm; Benjelloun Direct subm; Daga Direct subm; [1]; [2]; [3]; This Study
Central	102 (90.27)	…	…	…	…	11 (9.73)	…	113	[4]; Amills Direct subm; Githui Direct subm; [1]; [2]; [5]
Southern	89 (86.41)	14 (13.59)	…	…	…	…	…	103	Awotunde Direct subm; [1]; [2]; Wu and Liu Direct subm
**America**	**64 (100.00)**	…	…	…	…	…	…	**64**	
Central	34 (100.00)	…	…	…	…	…	…	34	[4]; Marcheco Direct subm
Southern	30 (100.00)	…	…	…	…	…	…	30	[4]
**Asia**	**2891 (84.93)**	**392 (11.52)**	**43 (1.26)**	**34 (1.00)**	**…**	**44 (1.29)**	**…**	**3404**	
Middle East	876 (93.99)	6 (0.64)	2 (0.21)	8 (0.86)	…	40 (4.29)	…	932	[6]; Akis Direct subm; Mazdarani Direct subm; Kiraz Direct subm; [1]; [2]; This Study; Wu and Liu Direct subm
Southern	830 (94.10)	31 (3.51)	9 (1.02)	11 (1.25)	…	1 (0.11)	…	882	Amills Direct subm; Das Direct sub]; Jimcy Direct subm; [7]; Karabasanavar Direct subm; [1]; [2]; Nomura et al Direct subm; Rana Direct subm; Sadia Direct subm; [8]; [9]; This Study
Eastern	1185 (74.53)	355 (22.33)	32 (2.01)	15 (0.94)	…	3 (0.19)	…	1590	Cai Direct subm; Chen and Wang Direct subm; [10]; Chi Direct subm; Fang Direct subm; Kim Direct subm; Li Direct subm; [11]; [12]; [13]; Liu Direct subm; [1]; [14]; Mannen Direct subm; [2]; Nomura et al Direct subm; Song and Lin Direct subm; Tang Direct subm; This Study; [15]; Wu and Liu Direct subm; Wu and Ma Direct subm; [16]; Xi Direct subm; Zhang Direct subm; [17]; Zhong and Wang Direct subm
**Europe**	**2148 (97.59)**	**2 (0.09)**	**45 (2.04)**	**3 (0.14)**	**3 (0.14)**	**…**	…	**2201**	
Continental	504 (95.27)	1 (0.19)	23 (4.35)	1 (0.19)	…	…	…	529	Amills Direct subm; Baldursdottir Direct subm; [18]; Daga Direct subm; [19]; [1]; Maudet and Taberlet Direct subm; [2]; [20]; This Study
Mediterranean	1644 (98.33)	1 (0.06)	22 (1.32)	2 (0.12)	3 (0.18)	…	…	1672	[4]; Amills Direct subm; [21]; Bigi Direct subm; Curik Direct subm; Daga Direct subm; [22]; Hughes Direct subm; [23]; [2]; [24]; [25]; [5]; [26]; This Study
**Total**	**5677 (90.51)**	**408 (6.51)**	**88 (1.40)**	**37 (0.59)**	**3 (0.05)**	**59 (0.94)**	**…**	**6272**	

Wild Goats

	Hg A	Hg B	Hg C	Hg D	Hg F	Hg G	Other	Total	References
Middle East	29 (6.35)	17 (3.72)	171 (37.42)	59 (12.91)	132 (28.88)	10 (2.19)	39 (8.53)	457	[1]; [27]; This Study
Southern Asia	…	…	14 (42.42)	…	6 (18.18)	…	13 (39.39)	33	[1]; [27]; [9]
Mediterranean Europe	4 (100.00)	…	…	…	…	…	…	4	This Study
**Total**	**33 (6.68)**	**17 (3.44)**	**185 (37.45)**	**59 (11.94)**	**138 (27.94)**	**10 (2.02)**	**52 (10.53)**	**494**	

Ancient Goats[b]

	Hg A	Hg B	Hg C	Hg D	Hg F	Hg G	Other	Total	References
**Asia**	**22 (88.00)**	**2 (8.00)**	**…**	**1 (4.00)**	**…**	**…**	**…**	**25**	
Middle East	15 (100.00)	…	…	…	…	…	…	15	[6]; [28]; [29]; [30]; [31]
Eastern	7 (70.00)	2 (20.00)	…	1 (10.00)	…	…	…	10	[32]
**Europe**	**23 (88.46)**	**1 (3.85)**	**2 (7.69)**	**…**	**…**	**…**	**…**	**26**	
Continental	…	1 (100.00)	…	…	…	…	…	1	[20]
Mediterranean	23 (92.00)	…	2 (8.00)	…	…	…	…	25	[19]; [33]; [34]
**Total**	**45 (88.24)**	**3 (5.88)**	**2 (3.92)**	**1 (1.96)**	**…**	**…**	**…**	**51**	

[a] Haplogroup frequencies (%) are in parentheses. Sequences with an ambiguous haplogroup affiliation and/or geographic origin were not included.

[b] Sequences reported by [18] were excluded due to their ambiguity: i) haplogroup affiliation was unclear, sequences did not match with known haplogroups probably because data derived from ancient parchments represent multiple individuals/species due to the complex stages of parchment manufacturing, ii) the geographic origin of the individuals may not match the location where the parchments have been stored.

Table S6. Diagnostic mutational motifs of goat mtDNA haplogroups and sub-haplogroups.

Clade	Diagnostic Coding Region Mutational Motif[a,b]
**A**	3194, 7839, 8055, 14746
**A1**	6186
**A1a**	9220
**A2**	194
**A2a**	7213
**A2a1**	7117
**A3**	4332, 10944
**A4**	4191
**A5**	6863
**A6**	11127
**A7**	8279, 11913
**B**	6096, 7342, 8635, 9010, 10813, 11702, 11872, 13178, 13286, 13681, 13762
**B1**	2849, 8703, 13964, 14461, 14795
**C**	1024, 1112, 1368, 4626, 8113, 8486, 9547, 9599, 10106, 10302, 10545, 10563, 10950, 11073, 11256, 11569, 11957, 12146, 12362, 12638, 13157, 15053
**C1**	165, 3599, 4569, 4707, 6390, 6537, 7657, 8300C, 8851, 9111, 9205, 9346, 11969, 12539, 12791, 14290, 14527
**C1a**	1191, 5775, 6324, 7117, 9061, 12386, 12626
**D**	4276, 7090, 7108, 8968, 11325
**D1**	2698, 3739, 6333G, 7324, 7463, 7910, 8474, 10001, 12014, 12242, 13293, 13894, 14317, 14500, 15238
**A'B'D'G**	203, 4032, 7052, 7660, 7885, 8480, 8730, 8818, 10479, 10686, 11364, 11691, 13100T, 13280, 15220
**A'D'G**	1849, 5601, 8078, 8959, 9826, 13741
**A'D**	2207, 3468, 10638, 12383, 13756, 15004
**A'B'C'D'G**	211, 2885C, 3002, 3131, 3293, 3770, 4713, 4890, 5712, 6093, 6414, 8264, 9994, 10356A, 10449, 12314, 13001, 13292, 13502, 14044, 14386, 14518
**G**	257, 726, 797, 3197, 3949, 4119, 8102, 8770, 9546, 11649, 12014, 12743, 14288, 14587, 15007, 15112
A.G.M.[c]	56, 100, 227, 289, 435, 945, 1075, 1829, 2268, 2311, 2663, 2922, 2978, 3248, 3261, 3491, 3635, 3960T, 4131, 4164, 4284, 4363, 4470, 4563, 4601A, 4749, 5085, 5141, 5312, 5658, 5772, 5791, 5850, 6150, 6309, 6486, 6528, 6923, 7024, 7151, 7318, 7361, 7426, 7642, 7669, 7842, 8456, 8597, 8668, 8758, 8827, 9007, 9085, 9086, 9139, 9223, 9297, 9346, 9445, 9470, 9572, 9677, 9753, 9797, 9935, 9937, 9943, 10229, 10261, 10374, 10416, 10430, 10440, 10473, 10557, 10776, 10842, 11001, 11052, 11163, 11797, 11849, 11912, 11915, 11973, 12044, 12218, 12257, 12260, 12369, 12651, 12683, 12762, 12776, 12987, 13146A, 13271, 13382, 13457, 13508, 13523, 13526, 13548, 13579, 13675, 13800, 14038, 14050, 14170, 14266C, 14284, 14341, 14444, 14524, 14593, 14650, 14674, 14686, 14720, 14746, 14767C, 14893, 14968, 14993, 15053, 15076, 15348

[a] Coding Region Mutational motifs are relative to the reference sequence (GRS, GenBank NC_005044.2), which is a member of haplogroup B.
[b] Recurrent mutations are underlined.
[c] Ancestral Goat Mitogenome.

Table S7. A comparison of the phylogeographic features of goat, taurine and horse mtDNA haplogroups identified by analyzing domestic breeds from Eurasia.

**Goat**[a]	***C. hircus***		**Taurine Cattle**[b]	***B. taurus***		**Horse**[c]	***E. caballus***	
**Hg**	**Age**	**Freq**	**Hg**	**Age**	**Freq**	**Hg**	**Age**	**Freq**
ABCDG *(A.d.G.M)*[d]	136.7		REPQT *(A.T.M.)*[e]	135.3		A-R *(A.M.M)*[f]	152.6	
A	12.8	89.9%	Q1	15.5	0.8%(Q)	A	3.2	6.6%
B1	14.2	7.0%	Q2	0.9	n.a.	B	7.1	8.2%
C1a	9.2	1.6%	R1	3.0	0.6%(R)	C	4.6	1.4%
D1	n.a.	0.7%	R2	n.a.	n.a.	D	n.a.	3.6%
G	9.0	0.8%	T1	9.4	5.8%	E	n.a.	0.9%
			T2	10.4	5.1%	G	6.6	10.3%
			T3	11.3	85.5%	H	n.a.	1.0%
			T5	10.2	0.7%	I	10.6	7.4%
						J	11.2	2.4%(J/K)
						K	n.a.	n.a.
						L	6.4	31.9%
						M	4.1	5.9%
						N	4.3	6.3%
						O	n.a.	n.a.
						P	4.5	3.5%(O/P)
						Q	10.1	6.9%
						R	3.0	1.9%

[a] Percentage frequencies among Eurasian domestic goats and age estimates are from this study.
[b] Percentage frequencies among domestic taurine breeds in Eurasia are from [35], except for Q, R and T5 [36]. ML age estimates (based on coding-region substitutions) are from [37], except for Q1, Q2 and R1 [36].
[c] Percentage frequencies and ML age estimates (based on synonymous substitutions) are from [38].
[d] Ancestral domestic Goat Mitogenome.
[e] Ancestral Taurine Mitogenome.
[f] Ancestral Mare Mitogenome.

Table S8. Oligonucleotides used to amplify and sequence (Sanger method) the goat mitochondrial genome.

PCR ID Number	Fragment Length (bps)	Oligonucleotide for PCR			Oligonucleotide for sequencing		
		Name[a]	Sequence (5'→3')	Tm (°C)	Name[a]	Sequence (5'→3')	Tm (°C)
**1**	1884	16539F	GCTCCCTCCTAGATATTAGTGCAA	59.47	16614F	TAAGTGCCTGGGTCTTTTAC	54.97
		1779R	GACTTAAACTTGTGCAGGGAGAAA	59.42	486F	CTGGGATTAGATACCCCACT	55.37
					1777R	CTTAAACTTGTGCAGGGAGA	55.28
**2**	1891	1601F	AGGCCTAAAAGCAGCCATC	57.80	1685F	CCTAGACCTAATACTGGACCAC	56.79
		3491R	ATAGGGGCTGTGAAATGCTC	57.64	2073F	CACGAGGGTTTTACTGTCTC	55.82
					2633F	TACAAACAAAACCTGCCCTA	54.76
**3**	1868	3128F	CATTGGAGCCTTACGAGCAG	58.71	3217F	CCTTCGCCCTCTCTACACTA	57.95
		4995R	CTTGCTTAGGGCTTTGAAGG	56.69	3793F	AAGAACCCAAAACTCTTCGT	55.11
					4261F	ACACAAGGTATCCCCCTATC	55.68
**4**	1907	4670F	CCCCACTATCAGGATTTGTACC	57.91	4736F	TCTTACCCACCCTTATAGCA	54,94
		6576R	AATTGCGAAGTGGATTTTGG	55.13	5253F	CAGGACTTGGTAAAAAGAGG	53.51
					5776F	CTACACCTAGCAGGCATCTC	57.19
**5**	1858	6221F	ACGTCGATACACGGGCTTAC	59.90	6313F	GGAGGCAATATCAAATGGT	52.58
		8078R	GAGTGCCCATTGTTGGAGAG	58.54	6840F	ATTTGAAGAACCCACATACG	53.60
					7401F	AACCTGGAGAACTACGACTG	56.60
**6**	1765	7772F	TGCCACAACTAGACACATCG	57.92	7864F	GACTTCTACCACAACCCAGA	56.48
		9536R	GGGGAGTCAAAATGCGATAG	56.29	8334F	CCACTAATCCCAATGCTAGT	54.50
					8806F	CTTCCAAGGTCATCATACTCC	55.47
**7**	1834	9208F	CACAGGATTCCACGGTCTTC	58.27	9288F	TCACCTCTAATCATCACTTCG	54.88
		11041R	TGACGGAAGAATATGCGATG	55.74	9773F	CGAATGAACTCAAAAAGGAC	52.83
					10332F	CTTCTTCTCCGACTCCCTAT	55.46
**8**	1769	10725F	ACCCAACTCCTGATCCAATG	57.19	10814F	TACCTAAAGCCCACGTAGAA	55.59
		12493R	GAGTGGAGTAATGCTGAAACGG	59.33	11258F	TAACTAATCTGGCCCTACCC	55.69
					11826F	TACAAATCCACCAACTACCC	54.64
**9**	1923	12123F	ATCACAATACTCATCCTCGTCACC	60.20	12215F	ATGGTGATATGGACGAACAG	54.94
		14045R	TCTTTGTAATAGGTTTTGTGGGGT	58.29	12709F	AACCCTACCTAGCATTCCTC	56.00
					13252F	TAGAAACCGCTAACATAACTCA	54.96
**10**	1871	13797F	TAACAAACGCCCCTAGAACAA	57.51	13852F	AGTAGCCATAGCCGTCGTATAACC	61.87
		15667R	GTAGGCGAGCGGTGTAATGTA	59.93	14221F	CCCATCAAACATCTCATCAT	52.99
					14779F	AGGAATTCCATCAGACGCAG	57.68
**11**	1634	15177F	CAGTCGAACATCCCTACATTATTATTGG	60.12	15306F	TACAATCAATACACTGGTCTT	52.18
		00167R	TTAGTCTTATTGATTTGGAGGGCGTTA	60.58	15935F	ATTAAACGATCTCCCCCAT	53.07

[a] Primer names correspond to the (5') nucleotide position in GRS.

Supporting references (*in extenso*):

1. Luikart G, Gielly L, Excoffier L, Vigne JD, Bouvet J, Taberlet P: **Multiple maternal origins and weak phylogeographic structure in domestic goats**. *Proc Natl Acad Sci U S A* 2001, **98**(10):5927-5932.
2. Naderi S, Rezaei HR, Taberlet P, Zundel S, Rafat SA, Naghash HR, el-Barody MA, Ertugrul O, Pompanon F, Consortium E: **Large-scale mitochondrial DNA analysis of the domestic goat reveals six haplogroups with high diversity**. *PLoS One* 2007, **2**(10):e1012.
3. Pereira F, Queirós S, Gusmão L, Nijman IJ, Cuppen E, Lenstra JA, Davis SJ, Nejmeddine F, Amorim A, Consortium E: **Tracing the history of goat pastoralism: new clues from mitochondrial and Y chromosome DNA in North Africa**. *Mol Biol Evol* 2009, **26**(12):2765-2773.
4. Amills M, Ramírez O, Tomàs A, Badaoui B, Marmi J, Acosta J, Sànchez A, Capote J: **Mitochondrial DNA diversity and origins of South and Central American goats**. *Anim Genet* 2009, **40**(3):315-322.
5. Royo LJ, Traoré A, Tambourá HH, Alvarez I, Kaboré A, Fernández I, Ouédraogo-Sanou G, Toguyeni A, Sawadogo L, Goyache F: **Analysis of mitochondrial DNA diversity in Burkina Faso populations confirms the maternal genetic homogeneity of the West African goat**. *Anim Genet* 2009, **40**(3):344-347.
6. Akis I, Oztabak K, Mengi A, Un C: **Mitochondrial DNA diversity of Anatolian indigenous domestic goats**. *J Anim Breed Genet* 2014, **131**(6):487-495.
7. Joshi MB, Rout PK, Mandal AK, Tyler-Smith C, Singh L, Thangaraj K: **Phylogeography and origin of Indian domestic goats**. *Mol Biol Evol* 2004, **21**(3):454-462.
8. Sultana S, Mannen H: **Polymorphism and evolutionary profile of mitochondrial DNA control region inferred from the sequences of Pakistani goats**. In: *Anim Sci J.* vol. 75; 2004: 303-309.
9. Sultana S, Mannen H, Tsuji S: **Mitochondrial DNA diversity of Pakistani goats**. *Anim Genet* 2003, **34**(6):417-421.
10. Chen SY, Su YH, Wu SF, Sha T, Zhang YP: **Mitochondrial diversity and phylogeographic structure of Chinese domestic goats**. *Mol Phylogenet Evol* 2005, **37**(3):804-814.
11. Lin BZ, Odahara S, Ishida M, Kato T, Sasazaki S, Nozawa K, Mannen H: **Molecular phylogeography and genetic diversity of East Asian goats**. *Anim Genet* 2013, **44**(1):79-85.
12. Liu RY, Yang GS, Lei CZ: **The genetic diversity of mtDNA D-loop and the origin of Chinese goats**. *Yi Chuan Xue Bao* 2006, **33**(5):420-428.
13. Liu YP, Cao SX, Chen SY, Yao YG, Liu TZ: **Genetic diversity of Chinese domestic goat based on the mitochondrial DNA sequence variation**. *J Anim Breed Genet* 2009, **126**(1):80-89.
14. Mannen H, Nagata Y, Tsuji S: **Mitochondrial DNA reveal that domestic goat (*Capra hircus*) are genetically affected by two subspecies of bezoar (*Capra aegagurus*)**. *Biochem Genet* 2001, **39**(5-6):145-154.
15. Wang J, Chen YL, Wang XL, Yang ZX: **The genetic diversity of seven indigenous Chinese goat breeds**. *Small Rumin Res* 2008, **74**:231-237.
16. Wu YP, Guan WJ, Zhao QJ, He XH, Pu YB, Huo JH, Xie JF, Han JL, Rao SQ, Ma YH: **A fine map for maternal lineage analysis by mitochondrial hypervariable region in 12 Chinese goat breeds**. *Anim Sci J* 2009, **80**(4):372-380.
17. Zhao Y, Zhao R, Zhao Z, Xu H, Zhao E, Zhang J: **Genetic diversity and molecular phylogeography of Chinese domestic goats by large-scale mitochondrial DNA analysis**. *Mol Biol Rep* 2014, **41**(6):3695-3704.
18. Campana MG, Bower MA, Bailey MJ, Stock F, O'Connell TC, Edwards CJ, Checkley-Scott C, Knight B, Spencer M, Howe CJ: **A flock of sheep, goats and cattle: ancient DNA analysis reveals complexities of historical parchment manufacture**. *J Archaeol Sci* 2010, **37**(6):1317-1325.
19. Fernández H, Hughes S, Vigne JD, Helmer D, Hodgins G, Miquel C, Hänni C, Luikart G, Taberlet P: **Divergent mtDNA lineages of goats in an Early Neolithic site, far from the initial domestication areas**. *Proc Natl Acad Sci U S A* 2006, **103**(42):15375-15379.
20. Schlumbaum A, Campos PF, Volkenc S, Volkenc M, Hafner A, Schibler J: **Ancient DNA, a Neolithic legging from the Swiss Alps and the early history of goat**. *J Archaeol Sci* 2010, **37**(6):1247-1251.
21. Azor PJ, Monteagudo LV, Luque M, Tejedor MT, Rodero E, Sierra I, Herrera M, Rodero A, Arruga MV: **Phylogenetic relationships among Spanish goats breeds**. *Anim Genet* 2005, **36**(5):423-425.
22. Doro MG, Piras D, Leoni GG, Casu G, Vaccargiu S, Parracciani D, Naitana S, Pirastu M, Novelletto A: **Phylogeny and patterns of diversity of goat mtDNA haplogroup A revealed by resequencing complete mitogenomes**. *PLoS One* 2014, **9**(4):e95969.
23. Martinez A, Ferrando A, Manunza A, Gomez M, Landi V, Jordana J, Capote J, Badaoui B, Vidal O, Delgado JV *et al*: **Inferring the demographic history of a highly endangered goat breed through the analysis of nuclear and mitochondrial genetic signatures**. *Small Rumin Res* 2012, **104**(1-3):78-84.
24. Pereira F, Pereira L, Van Asch B, Bradley DG, Amorim A: **The mtDNA catalogue of all Portuguese autochthonous goat (Capra hircus) breeds: high diversity of female lineages at the western fringe of European distribution**. *Mol Ecol* 2005, **14**(8):2313-2318.
25. Piras D, Doro MG, Casu G, Melis PM, Vaccargiu S, Piras I, Parracciani D, Stradoni R, Frongia B, Lai G *et al*: **Haplotype affinities resolve a major component of goat (*Capra hircus*) MtDNA D-loop diversity and reveal specific features of the Sardinian stock**. *PLoS One* 2012, **7**(2):e30785.
26. Sardina MT, Ballester M, Marmi J, Finocchiaro R, van Kaam JB, Portolano B, Folch JM: **Phylogenetic analysis of Sicilian goats reveals a new mtDNA lineage**. *Anim Genet* 2006, **37**(4):376-378.
27. Naderi S, Rezaei HR, Pompanon F, Blum MG, Negrini R, Naghash HR, Balkiz O, Mashkour M, Gaggiotti OE, Ajmone-Marsan P *et al*: **The goat domestication process inferred from large-scale mitochondrial DNA analysis of wild and domestic individuals**. *Proc Natl Acad Sci U S A* 2008, **105**(46):17659-17664.
28. Kahila Bar-Gal G, Ducos P, Kolska Horwitz L: **The application of ancient DNA analysis to identify neolithic caprinae: a case study from the site of Hatoula, Israel**. *Int J Osteoarchaeol* 2003, **13**:120-131.
29. Fernández H, Taberlet P, Mashkour M, Vigne JD, Luikart G: **Assessing the origin and diffusion of domestic goats using ancient DNA**. In: *The first steps of animal domestication.* Edited by Vigne J-D, Peters J, Helmer D. Oxford: Oxbow Books; 2005: 50-54.
30. Luikart G, Fernandez H, Mashkour M, England PR, Taberlet P: **Origins and Diffusion of Domestic Goats Inferred from DNA Markers Example Analyses of MtDNA, Y Chromosome, and Microsatellites**. In: *Documenting Domestication New genetic and archaeological paradigms.* Edited by Zeder MA, Bradley DG, Emshwiller E, Smith BD. Berkeley: University of California Press; 2006: 294-305.
31. Mazdarani FH, Akbari MT, Fard RMN, Hessari M, Pour KC: **Molecular identification of *Capra hircus* in east Chia Sabz, an Iranian pre-pottery Neolithic site, Central Zagros, based on mtDNA**. *J Anim Plant Sci* 2014, **24**(3):945-950.

32. Han L, Yu HX, Cai DW, Shi HL, Zhu H, Zhou H: **Mitochondrial DNA analysis provides new insight into the origins of Chinese domestic goat**. *Small Rumin Res* 2010, **90**:41–46.
33. Gerbault P, Powell A, Thomas MG: **Evaluating demographic models for goat domestication using mtDNA sequences**. *Anthropozoologica* 2012, **47**(2):65-78.
34. Hughes S, Fernández H, Cucchi T, Duffraisse M, Casabianca F, Istria D, Pompanon F, Vigne JD, Hänni C, Taberlet P: **A dig into the past mitochondrial diversity of Corsican goats reveals the influence of secular herding practices**. *PLoS One* 2012, **7**(1):e30272.
35. Lenstra J, Ajmone-Marsan P, Beja-Pereira A, Bollongino R, Bradley D, Colli L, De Gaetano A, Edwards C, Felius M, Ferretti L *et al*: **Meta-Analysis of Mitochondrial DNA Reveals Several Population Bottlenecks during Worldwide Migrations of Cattle**. *Diversity* 2014, **6**(1):178.
36. Bonfiglio S, Achilli A, Olivieri A, Negrini R, Colli L, Liotta L, Ajmone-Marsan P, Torroni A, Ferretti L: **The enigmatic origin of bovine mtDNA haplogroup R: sporadic interbreeding or an independent event of *Bos primigenius* domestication in Italy?** *PLoS One* 2010, **5**(12):e15760.
37. Achilli A, Bonfiglio S, Olivieri A, Malusa A, Pala M, Kashani BH, Perego UA, Ajmone-Marsan P, Liotta L, Semino O *et al*: **The multifaceted origin of taurine cattle reflected by the mitochondrial genome**. *PLoS One* 2009, **4**(6):e5753.
38. Achilli A, Olivieri A, Soares P, Lancioni H, Hooshiar Kashani B, Perego UA, Nergadze SG, Carossa V, Santagostino M, Capomaccio S *et al*: **Mitochondrial genomes from modern horses reveal the major haplogroups that underwent domestication**. *Proc Natl Acad Sci U S A* 2012, **109**(7):2449-2454.
